# Superpixel-Based Conditional Random Fields (SuperCRF): Incorporating Global and Local Context for Enhanced Deep Learning in Melanoma Histopathology

**DOI:** 10.3389/fonc.2019.01045

**Published:** 2019-10-11

**Authors:** Konstantinos Zormpas-Petridis, Henrik Failmezger, Shan E Ahmed Raza, Ioannis Roxanis, Yann Jamin, Yinyin Yuan

**Affiliations:** ^1^Division of Radiotherapy and Imaging, The Institute of Cancer Research, London, United Kingdom; ^2^The Royal Marsden NHS Trust, Surrey, United Kingdom; ^3^Division of Molecular Pathology, The Institute of Cancer Research, London, United Kingdom; ^4^Royal Free London NHS Foundation Trust, London, United Kingdom

**Keywords:** deep learning, machine learning, conditional random fields, digital pathology, cell classification, melanoma, tumor microenvironment

## Abstract

Computational pathology-based cell classification algorithms are revolutionizing the study of the tumor microenvironment and can provide novel predictive/prognosis biomarkers crucial for the delivery of precision oncology. Current algorithms used on hematoxylin and eosin slides are based on individual cell nuclei morphology with limited local context features. Here, we propose a novel multi-resolution hierarchical framework (SuperCRF) inspired by the way pathologists perceive regional tissue architecture to improve cell classification and demonstrate its clinical applications. We develop SuperCRF by training a state-of-art deep learning spatially constrained- convolution neural network (SC-CNN) to detect and classify cells from 105 high-resolution (20×) H&E-stained slides of The Cancer Genome Atlas melanoma dataset and subsequently, a conditional random field (CRF) by combining cellular neighborhood with tumor regional classification from lower resolution images (5, 1.25×) given by a superpixel-based machine learning framework. SuperCRF led to an 11.85% overall improvement in the accuracy of the state-of-art deep learning SC-CNN cell classifier. Consistent with a stroma-mediated immune suppressive microenvironment, SuperCRF demonstrated that (i) a high ratio of lymphocytes to all lymphocytes within the stromal compartment (*p* = 0.026) and (ii) a high ratio of stromal cells to all cells (*p* < 0.0001 compared to *p* = 0.039 for SC-CNN only) are associated with poor survival in patients with melanoma. SuperCRF improves cell classification by introducing global and local context-based information and can be implemented in combination with any single-cell classifier. SuperCRF provides valuable tools to study the tumor microenvironment and identify predictors of survival and response to therapy.

## Introduction

Cancer is a highly complex, non-autonomous disease. The interactions between microenvironmental selective pressures and cancer cells dictate how cancer progresses and evolves. Accurate and spatially explicit characterization of the tumor microenvironmental landscape including how cancer cells interact with the extra-cellular matrix and other cellular players such as stromal cells and immune cells within the tumoral niche, is needed to understand the context in which cancer evolves, and may also provide robust predictor of cancer behavior for risk-stratification (1). More specifically the recent success of cancer immunotherapy including the spectacular response observed in patients with previously incurable melanoma, a highly aggressive form of skin cancer, calls for a better understanding of the cancer-immune interface.

In the new era of digital pathology, advanced image analysis can objectively, consistently, and quantitatively characterize the different components of the tumor and how they spatially interact, and as a result assist pathologists in tasks such as tumor grading (2). Algorithms for cell detection and classification are key components of this process. Machine learning, and more recently deep learning algorithms, both exploiting the phenotypic differences in nuclear morphology between each cell type, revolutionized the field yielding significantly better cell detection, segmentation, and classification results (3–9).

However, even state-of-the-art deep learning algorithms can underperform especially in cases where different cell types appear morphologically similar. Current computed pathology tools focus on individual cell nuclei morphology with limited abstract local context features, whereas pathologists incorporate regional tissue architecture (in practice, by zooming in/out), together with cell morphological features to accurately classify cells.

**Figure 1:**
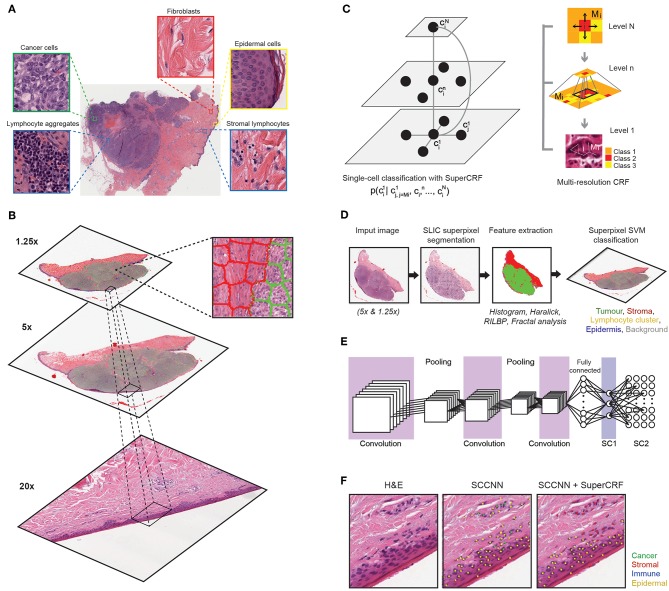
Overview of the SuperCRF framework for analyzing H&E-stained pathological images of melanoma. **(A)** Major histological features of melanoma architecture. **(B)** Projection of regional classification results using superpixels from various scales to the 20× magnification for the improvement of single-cell classification. **(C)** Graphical representation of node dependencies (cells and superpixels) across different scales. **(D)** Region classification scheme using a superpixel based machine-learning method in whole-slide images (5× and 1.25× magnification) **(E)** Single-cell classification using a state-of-the-art spatially constrained-convolution neural network (SC-CNN) classifier **(F)** representative results of the SC-CNN cell classifier alone and combined with our SuperCRF system. Note the misclassification of various stromal cells by the SC-CNN, which are corrected by our model.

Here, we hypothesize that robust tumor regional classification from lower resolution images can provide the contextual information that is key to further improve single cell classification algorithms. Our aim is to introduce dependencies on global tissue context and cell neighborhood and enhance learning results for cell classification from deep convolution neural networks (CNNs). Probabilistic graphical models have successfully been applied to improve cell classification in time-lapse imaging by taking into account the temporal context of a cell (10–15). Probabilistic graphical models have also been used successfully in histopathology images for pathology detection and segmentation (16–19), disease and tissue staging (20, 21), and nuclei segmentation (22). In our study, instead of time dependency, we apply graphical models to introduce the spatial context of a cell as additional information to improve single-cell classification. A multi-resolution hierarchical framework was proposed to mirror the way pathologists perceive tumor architecture, and applied to whole-slide images (WSI) hematoxylin and eosin (H&E)-stained slides of melanoma skin cancer (Figure 1A). We demonstrated that our new system is computationally efficient and significantly improves single cell classification. The increased accuracy in cell classification further enabled us to shed new light on the understanding of cancer-immune-stroma interface of melanoma.

## Materials and Methods

### Datasets

In total, 105 full-face, H&E stained section images from formalin-fixed, paraffin-embedded (FFPE) diagnostic blocks of melanoma skin cancer from The Cancer Genome Atlas (TCGA) were used. We scaled all digitized (Aperio ImageScope) histology images to 20, 5, and 1.25× magnification with pixel resolution 0.504, 2.016, and 8.064 μm, respectively, using Bio-Formats (https://www.openmicroscopy.org/bio-formats/). WSIs at 20× magnification (representative size: 30,000 × 30,000 pixels), were split into sub-images (tiles) of 2,000 × 2,000 pixels each, for computational efficiency.

For the purpose of training and testing the different parts of our system we divided the dataset into sub-datasets, namely single-cell classification dataset, 5× sub-dataset, 1.25× sub-dataset and discovery sub-dataset (Table 1, also see Supplementary Tables 1–4).

**Table 1:** Summary of the data used to train and test the different parts of the SuperCRF system, as well as study the cancer-immune-stroma interface (also, see Supplementary Tables 1–4).

**Name**	**Number of WSIs**	**Purpose**
**Single-cell** classification sub-dataset	**8** Training SC-CNN: 3 (348 tiles) Training SuperCRF: 2 (84 tiles) Testing: 3 (290 tiles)	Single-cell classification into four categories: cancer cells, lymphocytes, stromal cells, epidermal cells
**5x** sub-dataset	**16** Training: 10 Testing: 6	Region classification into five categories: tumor, normal stroma, lymphocyte cluster, normal epidermis, lumen/white space
**1.25x** sub-dataset	**58** Training: 21 Testing: 37	Region classification into four categories: tumor, normal stroma, normal epidermis, lumen/white space
**Discovery** dataset	**97**	Study of the tumor-stroma interface. To accelerate the analysis, 50 tiles (2,000 × 2,000 pixels) containing tumors were randomly sampled from every whole-slide image (WSI)

*The values are bold for visual (illustration) purposes*.

### Single-Cell Classification Using a Spatially Constrained Convolutional Neural Network

We used a Spatially Constrained Convolutional Neural Network (SC-CNN) (6) for single cell classification (Figure 1E). SC-CNN uses spatial regression in order to predict the probability of a pixel being the center of the nucleus. The nucleus is classified by a neighboring ensemble predictor (NEP) in conjunction with a standard softmax CNN. We randomly initialized the network's layers as we have found that to perform better than transfer learning from real-world datasets in our experiments with pathological samples.

### Superpixel-Based Tumor Region Classification

A machine learning superpixel-based framework was implemented in Matlab (23) to classify tumor tissue regions and was subsequently applied to low resolution (5 and 1.25×) images. Reinhard stain normalization (24) was applied separately on each of the 5 and 1.25× sub-datasets to account for stain variabilities that could affect the classification (25).

Downscaled images were segmented using the simple linear iterative clustering (SLIC) superpixels algorithm (26), which is designed to provide roughly uniform superpixels. Choosing the optimal number of superpixels is important to ensure that the superpixels capture homogeneous areas and adhere to image boundaries. With our pathologist's input, we visually identified a size of superpixels that met these criteria and chose the number of superpixels automatically based on each image's size (Equation 1).

(1) $$\begin{array}{l} N_{i}=ceiling\;\left(\frac{S_{i}}{U}\right) \end{array}$$

where *N*_*i*_ is the number of superpixels in the *i*th image, *S*_*i*_ is the size of image *i* in pixels, and *U* (here *U* = 1,250) is a constant held across all images that defined a desired size of the superpixels. This means, on average, a superpixel occupies an area of approximately 35 × 35 pixels, equivalent to 280 × 280 mm^2^. We identified the superpixels belonging to each area by determining whether their central points fell within the regions annotated by the pathologist.

Overall, for the 1.25× training sub-dataset, we found 15,477 superpixels belonging in tumor areas, 6,989 in stroma areas, 141 in epidermis and 691 in lumen/white space, while for the 5× training sub-dataset we found 1,193 superpixels belonging in tumor areas, 1,324 in stroma areas, 360 in epidermis, 506 in lymphocyte clusters and 830 in lumen/white space.

Next, we extracted four types of features, 85 in total, from each superpixel, including seven histogram features (mean values of hue, saturation, and brightness, sum of intensities, contrast, standard deviation, and entropy), and well-established texture features [12 Haralick features (27), 59 rotation-invariant local binary patterns (RILBP), 7 segmentation-based fractal texture analysis (SFTA) features (28)]. Features were standardized into *z*-scores. The mean values and standard deviation of the features from the training set were used for the normalization of the test set. A support vector machine (SVM) with a radial basis function (RBF, γ = 1/number_of_features) was trained with these features to classify superpixels into different biologically meaningful categories.

For the 5× sub-dataset, superpixels were classified into five categories: tumor area, normal stroma, normal epidermis, lymphocytes cluster, and lumen/white space. We increased the penalty in the cost function for the epidermis and lumen/white space classes by a factor of 10 when training the SVM, to account for class imbalance. For the 1.25× sub-dataset superpixels classification consisted of four categories: tumor area, normal stroma, normal epidermis, and lumen/white space. We randomly selected a subset of 5,000 cancer and stroma superpixels and increased the penalty in the cost function for the epidermis and lumen/white space classes by a factor of 10, again to account for class imbalance (Figure 1D).

### SuperCRF

Single-cell based classification approaches often assign a class label based on the morphology of -individual cells, regardless of their neighboring cells. However, these spatial relationships provide important information that is used by pathologists. Conditional random fields (CRF) are undirected graphical models that represent efficient ways to model dependences, by factorizing the probability density into a specific set of conditional dependence (29). Therefore, the tumor microenvironment can be modeled by a CRF by introducing nodes for cells and superpixels, as well as edges whenever there is a spatial relationship between nodes.

We excluded lymphocytes from the CRF assumption that neighboring cells have a higher probability to share the same class labels, since they infiltrate, in an inconsistent manner ranging from sparse to highly dense, in tumor as well as stromal tissue. Therefore, lymphocytes kept their label as assigned by the SC-CNN.

Let *n* be the total number of cells (besides lymphocytes) in the image and *c*_*i*_∈{*stromal, cancer, epidermis*}, *i* = 1, 2, …, *n* the input labels of the cells as assigned by the SC-CNN. Let *s*_*i*_, be the corresponding superpixel for a cell *c*_*i*_ with *s*_*i*_∈{*stromal, cancer, epidermis, white space*} for 1.25× superpixels and *s*_*i*_∈{*stromal, cancer, epidermis, lymphocyte, whitespace*} for 5× superpixels. **x**∈{**c**, **s**} comprises the nodes of the CRF. The CRF assigns output labels *y*_*i*_∈{*stromal, cancer, epidermis, lymphocyte, white space*} based on the input data. The joint probability distribution over input data and output labels, *p*(*y*_1_, *y*_2_, …, *y*_*n*_ ⌊*x*_1_, *x*_2_, …, *x*_*n*_) can be modeled by factorizing the probability density into a specific set of conditional dependence relationships (Figure 1C).

(2) $$\begin{array}{l} p\left(y|\;x\right)=\overset{i=1}{\underset{n}{\prod }}p\left(y_{i}\;|x_{i}\;\right)=\;\frac{1}{Z}\text{ exp}\left(\sum E\left(x_{i},y_{i},\;xN_{i},\;yN_{i}\right)\right) \end{array}$$

where *Z* is a normalizing constant, w is a weight vector and

(3) $$\begin{array}{l} E\left(x_{i},y_{i},\;xN_{i},\;yN_{i},\right)=\;\sum \Phi \left(x_{i},y_{i}\right)+\sum \psi_{c}\left(xN_{i},\;yN_{i}\right) \end{array}$$

defines the energy function of the CRF.

The node potentials Φ(*x*_*i*_, *y*_*i*_) represent the evidence that a cell *i*, with the input label *x*_*i*_ takes the class label *y*_*i*_. The node potential can be defined as Φ(*x*_*i*_, *y*_*i*_) = *f*(*x*_*i*_, *y*_*i*_)+b, with $f\left(x_{i,}y_{i}\right)=\{\begin{array}{lll} 1 & \; & if\;y_{i}=x_{i}\\ 0 & \; & else \end{array}$ and *b* representing the bias.

The edge potentials ψ_*c*_(*xNi, yNi*) model the probability that neighboring cells take a similar cell label. *N*_*i*_ is the neighborhood of cell *i*, defined as all the cells that can be found in a defined distance. The edge potentials are defined as: ψ_*c*_(*x*_*i*_, *y*_*i*_, *xN*_*i*_, *yN*_*i*_) = *f*(*x*_*i*_, *y*_*i*_)*f(*xN*_*i*_, *yN*_*i*_)+*b*.

The CRF was trained with stochastic gradient descent and the decoding was applied using loopy belief propagation. The toolbox of M. Schmidt was used to train and decode the CRF (30).

The source code for the study is available at Github (https://github.com/Henrik86/SuperCRF).

### Survival Analysis

We evaluated the prognosis value of the abundance of stromal cells and location of lymphocytes in our discovery sub-dataset. The ratio of stromal cells to all cells, the ratio of lymphocytes in cancer areas to all lymphocytes, and the ratio of lymphocytes in stroma areas to all lymphocytes were calculated for each patient. Patients were divided into high and low ratio groups, split at the median value of all scores. Patients with a ratio of lymphocytes being high inside the tumor area and low in the stroma were categorized as the “immune infiltration” group whereas patients with a ratio of lymphocytes being low in the tumor area and high in the stroma were categorized as “immune excluded,” based on the recent classification of the main immune phenotypes of anticancer immunity that predict response to immunotherapy (31). The number of patients belonging to neither of these two groups (high/high *n* = 6 and low/low *n* = 5) was too small to perform the survival analysis. Non-parametric Kaplan-Meier estimation was used to analyze overall survival in 94 patients. Differences between survival estimates were assessed with the log-rank test. Finally, Cox regression models were adjusted, testing for the independent prognostic relevance of our risk scores. To test if Breslow-thickness (the distance between the upper layer of the epidermis and the deepest point of tumor penetration) was contributing to a high ratio of stromal cells, we created a multivariate model containing both stromal cells ratio and Breslow-thickness, as well as two univariate models containing the covariates separately. Pearson's correlation was used to test for linear relation between the two variables.

## Results

### SuperCRF Improves Accuracy of Cell Classification

First, we trained the state-of-the-art deep learning method, spatially-constrained CNN (SC-CNN) algorithm, to detect and classify cells in high resolution (20×) WSI into four categories: cancer cells, stroma cells, lymphocytes, and epidermis cells. The SC-CNN network yielded an accuracy of 84.63% over 4,059 cells in the independent test set (Table 1, Supplementary Table 5). Visual inspection revealed that the majority of false positives were misclassification of stromal and cancer cells as epidermis, which confirmed our initial motive for the incorporation of regional and spatial information to improve classification.

**Figure 2:**
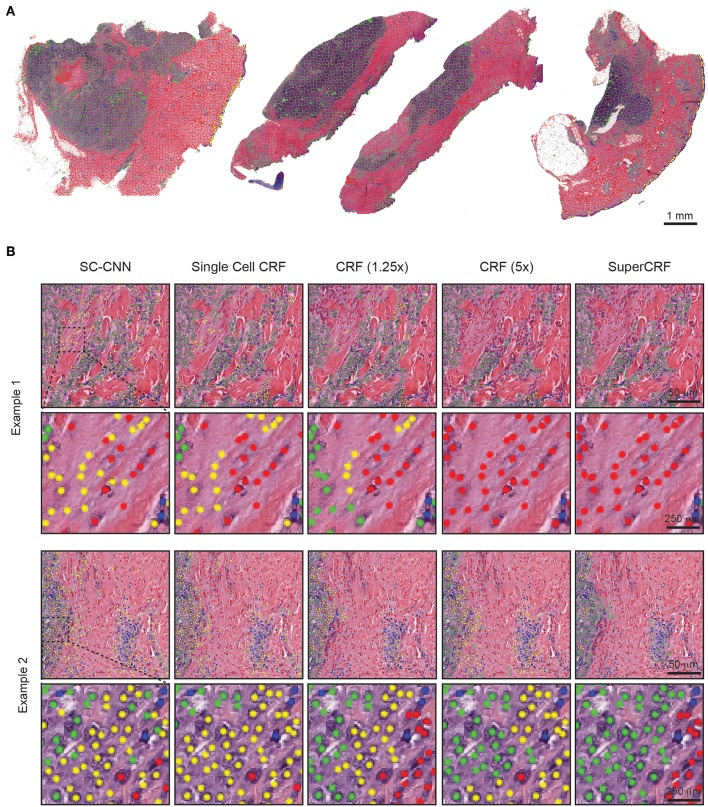
Representative examples of both superpixel and single-cell classification with or without SuperCRF. **(A)** Superpixels-based regional classification on representative whole slide images (5× magnification) of melanoma. Green: tumor area, Red: stroma area, Blue: normal epidermis, Yellow: lymphocyte cluster. **(B)** Representative images showing cell classification using a state-of-the-art spatially constrained-convolution neural network (SC-CNN) and four conditional random fields (CRF) models. Note the mislabeling of many cancer and stromal cells as epidermis cells when using the SC-CNN and the gradual increase in classification accuracy with the best accuracy achieved with the SuperCRF. Green, cancer cells; Red, stromal cells; Blue, lymphocytes; Yellow, epidermis cells.

Subsequently, we trained a conditional random field (CRF) by combining the cellular neighborhood with tumor region classification (cancer area, normal stroma, normal epidermis, lymphocyte cluster, and lumen/white space) from low resolution images (5 and 1.25×, Figure 1B), given by the superpixel-based machine-learning framework. The SLIC superpixels algorithm has previously been shown to be computationally efficient, requiring only 3s on average to segment a single downscaled image of 2,500 × 2,500 pixels using a 2.9 GHz Intel core i7 processor. Performance of classification using individual and various combinations of feature sets was tested and the use of all 85 features, yielded the highest accuracy (23). It was then applied on the two datasets of 1.25 and 5× magnification (Figure 2A) and achieved high accuracy in regional classification (1.25× sub-dataset: Overall accuracy 97.7% in the training set using 10-fold cross validation and 95.7% in 2,997 superpixels annotated in the 37 images of the independent test set; 5× sub-dataset: Overall accuracy 97.1% in the training set using 10-fold cross validation and 95.2% in 1,798 superpixels annotated in the six images of the independent test set).

To train SuperCRF, we first introduced dependencies on cell neighborhood. Cells were considered neighbors in the CRF, if they were located in a spatial proximity of 15 μm (or 30 pixels), which resulted in an average of 1.3 neighbors per cell. Subsequently, we integrated this local neighborhood with global context by connecting the CRF single-cell nodes to the regional classification results from superpixels. To determine the best configuration, we trained four different CRFs and compared their performance in terms of single-cell classification on a test set, including three samples, 290 tiles and 4,059 single-cell annotations (1,527 cancer cells, 676 lymphocytes, 837 normal epidermis cells, 1,019 stromal cells).

In detail, for the first CRF we did not use any context classification, just cell neighborhood dependencies, i.e., the only edges of the CRF were between neighboring cells (singleCellCRF). For the second and third CRF we introduced superpixel nodes. Now, single-cell nodes are not only connected to neighboring cells but every single-cell node is also connected to a superpixel node. We trained a CRF for 5× superpixel classification (CRF5×) and 1.25× superpixel classification (CRF1.25×). Furthermore, we trained a CRF in which every single-cell node was connected to two superpixel nodes in 5 and 1.25× resolution (SuperCRF). Already the singleCellCRF (Accuracy: 87.6%, Precision: 89.7%, Recall: 89.5%, Table 2) improves the classification accuracy compared to the SC-CNN (84.6%, Precision: 87.6%, Recall: 88.1%, Table 2). However, the use of contextual information by the introduction of superpixel nodes, markedly improves the classification metrics (Accuracy 90.8%, Precision: 92.5%, Recall: 91.1%, Table 2) for CRF1.25× and (Accuracy 91.7%, Precision: 93%, Recall: 91.3%, Table 2) for the CRF5×. The SuperCRF, using nodes from superpixels in both 5 and 1.25× resolution images, as well as the neighboring cells, resulted in the highest classification outcome (Accuracy 96.5%, Precision: 96.4%, Recall: 96.3%, Table 2, Figures 1F, 2B, Supplementary Tables 5–9).

**Table 2:** Evaluation of different conditional random fields (CRF) versions and a state-of-the-art spatially constrained-convolution neural network (SC-CNN) deep learning cell-classifier.

**Method**	**Accuracy (%)**	**Precision**	**Recall**
SC-CNN	84.63	0.8756	0.8808
singleCellCRF	87.61	0.8973	0.8946
CRF1.25×	90.79	0.9248	0.9110
CRF5×	91.70	0.9298	0.9126
SuperCRF	**96.48**	**0.9644**	**0.9629**

*The values are bold to indicate the highest achieved accuracy, precision and recall*.

### SuperCRF's Increased Accuracy of Cell Classification Improves Confidence in Stromal Cell Ratio as a Predictive Feature of Survival in Melanoma

**Figure 3:**
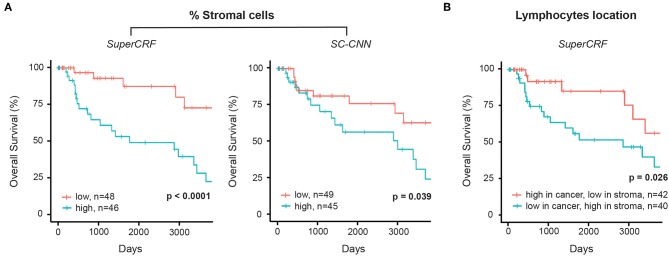
Associations between survival outcomes and SuperCRF-define risk groups in the Cancer Genome Atlas (TCGA) cohorts of patients with melanoma. **(A)** Kaplan-Meier Survival curves for patients in the high-risk group (blue) and low risk group classified by stromal cells ratio derived from SuperCRF (left) and using only the SC-CNN classifier. Note the difference in the p-value using the two methods. **(B)** Kaplan-Meier Survival curves for patients in the high-risk group (blue) and low risk group classified by immune phenotype based on spatial distribution of lymphocytes in different tumor compartments derived from SuperCRF.

The crosstalk between cancer cells and stromal cells play an active role in tumor invasion and metastasis, and controlling immune infiltration and is increasingly recognized as a hallmark of cancer (32). Tumor-stromal cell ratio has been shown to hold prognostic and predictive information in patient with solid tumors (31, 33, 34). Here, we demonstrate that a high stromal cell ratio is also a predictor of poor prognosis in melanoma using both values derived from the multivariate models of SC-CNN and SuperCRF in our discovery sub-dataset. Yet SuperCRF yields a significantly higher confidence in the predictive value of the stromal cell ratio (SuperCRF: *p* < 0.0001, Coxph-Regression (discretized by median): HR = 4.1, *p* = 0.006; SC-CNN: *p* = 0.039, Coxph-Regression (discretized by median): HR = 2.4, *p* = 0.05, Figure 3A).

Similar regression coefficients for both stromal cells ratio and Breslow-thickness covariates were observed between the multivariate and the two univariate survival models (1.404 and 0.171, respectively, for the multivariate model and 1.633 and 0.179 for the univariate models) of the SuperCRF. Pearson's correlation showed no correlation between stromal cells ratio and Breslow-thickness (*r* = −0.05), overall indicating that stromal cells ratio is independent to Breslow-thickness.

### Combining Cell and Region Classification: Location of the Immune Infiltrate Is Predictive of Survival in Melanoma

There is increasing evidence of the value of immune infiltration to provide prognostic information and predictors of response in patient with melanoma [recently reviewed in (35)]. The spatial compartmentalization of immune cells afforded by our SuperCRF (by the cell and region classification results) was used to define the recently-described main immune phenotypes of anticancer immunity that predict response to immunotherapy (31). Patients with a classified “immune excluded” phenotype, defined by a low lymphocyte ratio inside the tumor area and high inside the stroma area, was associated with a significantly worse prognosis compared to “inflamed” tumors characterized by a high ratio of lymphocytes inside the tumor and a low ratio inside the stroma (*p* = 0.026, Cox PH –regression: HR = 2.57, *p* = 0.032, Figure 3B). Taken together, our data is consistent with the model of a stroma-mediated immune suppressive microenvironment that exclude T cells from the vicinity of cancer cells.

## Discussion

In this study, we implemented a framework which fuses traditional machine learning with deep learning to model the way pathologists incorporate large-scale tissue architecture and context across spatial scales, to improve single-cell classification in large whole-section slide images. Using this approach, we demonstrated a marked 11.85% overall improvement in the accuracy of the state-of-art deep learning SC-CNN cell classifier. Also, the similar values of both precision and recall and their simultaneous increase in every step show the unbiased nature of our approach.

Computational pathology algorithms, typically exploit the inter-cell phenotypic differences for cell classification, yet even state-of-art deep learning algorithms tend to underperform in this task, mainly due to the disproportional numbers of cells sharing similar nuclear morphological features, or due to intra-class diversity, seen for example in tumor stroma (fatty tissue, necrosis, vessels, muscle, fibroblasts, and associated collagen). Whilst computers can quantify morphological differences in a considerably more complex way, pathologists still generally outperform computers in cell classification. An essential reason is that they incorporate key contextual information such as heterogeneous tissue architecture, together with cell morphological features.

The idea that a cancer cell is dependent on its neighboring cells and global context is comparable to the fundamental concept in landscape ecology that a living population depends on the existing habitats and is not equally spread on the terrain. A particular habitat could favor the development of specific organisms. In practice, landscape ecologists denote the habitats from satellite images and then “ground-truth” them by detailed small-scale sampling of the habitats of interest (36). This inspired the design of our framework by introducing CRF dependencies between (i) the cells and their neighbors and (ii) the cells and to the global context (i.e., habitats from low resolution captured by the classified superpixels).

Our proposed framework connects deep learning and classical image processing using probabilistic graphical models. All the information was combined using a CRF graphical model, which have been widely applied in image analysis for pathological images, yet mainly for semantic segmentation (16, 17, 37, 38). Here, (1) we introduce a new way to capture high-level spatial context using superpixels, (2) propose a new CRF model that introduces dependences over space and across different spatial scales, thereby modeling multiple cells and their associated superpixels simultaneously for more accurate classification, (3) introduce the concept of context-specific CRF modeling, given that the strength of dependence can be variable according to tumor compartments. There is an increasing interest in combining deep learning with different strategies, or “umbrella approaches,” such as the use of traditional machine learning to spatially explicit context used in this study, with the aim to, not only refine and improve the overall existing deep learning network (17, 39–41), but also facilitate biological interpretation compared to the “black-box”-like approach of deep-learning-only methods. However, optimizing and inventing new and refined deep learning networks is of equal importance, as during experimentation we observed that the better we made our single-cell classifier baseline, the more effective our SuperCRF approach became.

We also showed that combining cell classification with the global context given by the region classification (both inherent parts of the SuperCRF architecture) can open new avenues to study the cancer microenvironment from histopathological slides. For example, the spectacular response observed in clinical trials of immunotherapy in patients with incurable melanoma calls for a better understanding of the tumor microenvironment and in particular the cancer-immune-stroma interface. Here, our approach and its ability to look at lymphocytes within their cellular and global context can predict melanoma patient survival and potentially provide biomarker stratification for immunotherapeutic approaches, by identifying the three main types of tumor immunophenotypes including (i) inflamed tumors which are characterized by infiltrated T Cells within the tumor, and associated with a generally good prognosis (ii) immune-excluded tumors, in which T cells are present but prevented to infiltrate the tumor due to stromal interaction, and associated with worse prognosis (and obviously (iii) immune desert tumors). This could also potentially be extended to provide quantitative biomarkers to characterize the immune infiltrating response to immunotherapy. We also demonstrated that in accordance with the immune-excluded phenotype, tumors rich in stromal cells had a marked poorer prognosis in patients with melanoma. With *p*-value lower by two orders of magnitude, our method provide stronger predictive power than by using deep-learning only method for cell classification.

In the future, we plan to extend our framework and include an upward optimization step for the superpixels which may include additional classes for cells, regions and structures in order to provide a complete characterization of the tumor microenvironment. This may include deriving further classes from higher resolution images as we did for lymphocyte clusters in this study which were difficult to visualize in 1.25× resolution images. Incorporating additional deep learning methods should also be explored to perfect the classification of superpixels, for example by incorporating features extracted from a DCNN or a deep autoencoder, or to provide a potential alternative to superpixels, which may not be appropriate for the characterization of complicated structures, such as glands (42).

The primary aim of this study was to demonstrate proof-of-principle that the introduction of global and local context as cell dependencies using a probabilistic graphical model as a post-processing step, like an “umbrella,” can significantly improve the performance of deep learning or classical machine learning cell classifiers based only on cell-morphology and abstract local context information. We chose the SC-CNN architecture as our primary cell classification step due to its state-of-the-art performance in cell detection and classification compared to other well-established deep learning and classical machine learning approaches (6). Alternatively, other promising deep learning networks could potentially be used including Inception v3 (43), Inception v4 (44), or a VGG architecture (45).

Overall, our vision is to establish a network which will provide a complete characterization of every component of the tumor microenvironment where all the parts will interact with each other like an ecological landscape. Such system has immense potential and can be virtually transferred to any cancer type, to provide a better understanding of the cancer-immune cell interface, cell-stroma interactions, and predictive biomarkers of response to novel therapies, including immunotherapy, which has radically changed melanoma patient survival.

## Conclusion

The novel general framework SuperCRF improves cell classification by introducing global and local context-based information much like pathologists do. SuperCRF can be implemented in combination with any single-cell classifier and represent valuable tools to study the cancer-stroma-immune interface, which we used to identify predictors of survival in melanoma patients from conventional H&E stained histopathology.

## Data Availability Statement

Publicly available datasets were analyzed in this study. This data can be found here: https://www.cancer.gov/about-nci/organization/ccg/research/structural-genomics/tcga.

## Author Contributions

KZ-P, HF, SR, IR, YJ, and YY: substantial contributions to the conception or design of the work; or the method development, analysis, or interpretation of data for the work; drafting the work or revising it critically for important intellectual content; final approval of the version to be published; agreement to be accountable for all aspects of the work in ensuring that questions related to the accuracy or integrity of any part of the work are appropriately investigated and resolved.

### Conflict of Interest

The authors declare that the research was conducted in the absence of any commercial or financial relationships that could be construed as a potential conflict of interest.
