# Supplementary material for: Superpixel-Based Conditional Random Fields (SuperCRF): Incorporating Global and Local Context for Enhanced Deep Learning in Melanoma Histopathology
Source: Front Oncol. 2019 Oct 11;9:1045. doi: 10.3389/fonc.2019.01045 (PMC6798642; doi:10.3389/fonc.2019.01045)
Supplement: Supplementary file 5 [file Table_5.DOCX]

|  | | **SC-CNN** | | | |
| --- | --- | --- | --- | --- | --- |
|  |  | **C** | **E** | **L** | **S** |
| **Classes**  **(Cells)** | **Cancer** | 1149 | 2 | 0 | 11 |
|  | **Epidermis** | 348 | 834 | 16 | 204 |
|  | **Lymphocytes** | 24 | 1 | 660 | 12 |
|  | **Stromal** | 6 | 0 | 0 | 792 |

**Supplementary Table 5** – Confusion matrix of the classified cells from the spatially constrained-convolution neural network (SC-CNN) deep learning cell-classifier. C: cancer cells, E: epidermis cells, L: lymphocytes, S: Stromal cells.
