# Supplementary material for: Superpixel-Based Conditional Random Fields (SuperCRF): Incorporating Global and Local Context for Enhanced Deep Learning in Melanoma Histopathology
Source: Front Oncol. 2019 Oct 11;9:1045. doi: 10.3389/fonc.2019.01045 (PMC6798642; doi:10.3389/fonc.2019.01045)
Supplement: Supplementary file 6 [file Table_6.DOCX]

**Supplementary Table 6** – Confusion matrix of the classified cells from singleCellCRF architecture, where the trained conditional random field (CRF) combines the classified cells from the spatially constrained-convolution neural network (SC-CNN), with local context information (neighboring classified cells). C: cancer cells, E: epidermis cells, L: lymphocytes, S: Stromal cells.

|  | | **singleCellCRF** | | | |
| --- | --- | --- | --- | --- | --- |
|  |  | **C** | **E** | **L** | **S** |
| **Classes**  **(Cells)** | **Cancer** | 1263 | 0 | 1 | 17 |
|  | **Epidermis** | 236 | 836 | 12 | 197 |
|  | **Lymphocytes** | 24 | 1 | 660 | 12 |
|  | **Stromal** | 4 | 0 | 3 | 793 |
