# Supplementary material for: Superpixel-Based Conditional Random Fields (SuperCRF): Incorporating Global and Local Context for Enhanced Deep Learning in Melanoma Histopathology
Source: Front Oncol. 2019 Oct 11;9:1045. doi: 10.3389/fonc.2019.01045 (PMC6798642; doi:10.3389/fonc.2019.01045)
Supplement: Supplementary file 8 [file Table_8.DOCX]

**Supplementary Table 8** – Confusion matrix of the classified cells from CRF5x architecture, where the trained conditional random field (CRF) combines the classified cells from the spatially constrained-convolution neural network (SC-CNN), with the region classification information from the 5x magnification whole-slide images. C: cancer cells, E: epidermis cells, L: lymphocytes, S: Stromal cells.

|  | | **CRF5x** | | | |
| --- | --- | --- | --- | --- | --- |
|  |  | **C** | **E** | **L** | **S** |
| **Classes**  **(Cells)** | **Cancer** | 1311 | 2 | 2 | 6 |
|  | **Epidermis** | 177 | 778 | 1 | 28 |
|  | **Lymphocytes** | 24 | 1 | 660 | 12 |
|  | **Stromal** | 15 | 56 | 13 | 973 |
